# Determination of plane stress state using terahertz time-domain spectroscopy

**DOI:** 10.1038/srep36308

**Published:** 2016-11-08

**Authors:** Zhiyong Wang, Kai Kang, Shibin Wang, Lin'an Li, Ningning Xu, Jiaguang Han, Mingxia He, Liang Wu, Weili Zhang

**Affiliations:** 1 Department of Mechanics, School of Mechanical Engineering, Tianjin University, Tianjin, 300072, China; 2 School of Electrical and Computer Engineering, Oklahoma State University, Stillwater, Oklahoma, 74078, USA; 3 Center for Terahertz Waves and College of Precision Instrument and Optoelectronics Engineering, Tianjin University, Tianjin, 300072, China

## Abstract

THz wave has been increasingly applied in engineering practice. One of its outstanding advantages is the penetrability through certain optically opaque materials, whose interior properties could be therefore obtained. In this report, we develop an experimental method to determine the plane stress state of optically opaque materials based on the stress-optical law using terahertz time-domain spectroscopy (THz-TDS). In this method, two polarizers are combined into the conventional THz-TDS system to sense and adjust the polarization state of THz waves and a theoretical model is established to describe the relationship between phase delay of the received THz wave and the plane stress applied on the specimen. Three stress parameters that represent the plane stress state are finally determined through an error function of THz wave phase-delay. Experiments were conducted on polytetrafluoroethylene (PTFE) specimen and a reasonably good agreement was found with measurement using traditional strain gauges. The presented results validate the effectiveness of the proposed method. The proposed method could be further used in nondestructive tests for a wide range of optically opaque materials.

Terahertz (THz) wave has obtained wide applications in various fields since powerful sources were developed^1^,^2^. As a key spectral analysis technique in the THz waveband, THz-TDS obtained many valuable applications in characterization of semiconductors and biological materials^3^,^4^,^5^,^6^, 2D and tomographic imaging^7^,^8^, and geometric parameter measurement^9^. One outstanding feature of the THz wave is its high transmission ability through most optically opaque materials^10^,^11^,^12^, so that it can be used to determine and analyze the internal properties of these materials.

Stress state is important interior information of optically opaque materials as a response to external mechanical loadings. Recently, researchers reported their progresses in studying the stress-optical effect in THz regime^13^,^14^,^15^,^16^,^17^. In order to clarify the subtle differences among these works, a brief introduction to the stress-optical law is presented. The stress-optical law states that mechanical stress makes an originally isotropic material become optically anisotropic and the optical axes of the stress induced birefringence are aligned with the stress principle axes. If the specimen is under the plane stress state, the refractive index variations can be expressed using the stress parameters according to the following equation^18^


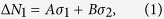



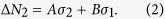


where Δ*N*_1_ and Δ*N*_2_ are the variations in material refractive index along the directions of the principle stresses, and *A* and *B* are called stress-optical coefficients. For a plane stressed specimen, three parameters, including *σ*_1_, *σ*_2_ and *θ*, are needed to describe its stress state. Here, *σ*_1_ and *σ*_2_ are the so-called principle stresses, while *θ* is the angle between the *x*-axis and *σ*_1_. Apparently, the plane stress state can be determined if Δ*N*_1_ and Δ*N*_2_, and *θ* are obtained in advance. Subtract Eq. (2) from Eq. (1), it can be obtained that


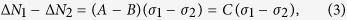


where parameter *C* is the stress-optical coefficient used in the photoelasticity and *C* = *A* − *B*.

To the best knowledge of the authors, the research work on stress measurement using THz radiation was launched in 2004 by Tsuguhiro^13^. An experimental system was built based on back-ward wave oscillator to carry out experiments on a polyethylene specimen. The difference between Δ*N*_1_ and Δ*N*_2_ was measured and the stress-optic coefficient *C* was obtained. By using a movable loading stage, the intensity distribution of the transmitted THz wave was also obtained when the polarizer and analyzer formed a cross-Nicol system. However, the stress information was not extracted from the intensity distribution. Ebara *et al*. constructed a polarized sensitive TDS system that could detect small birefringence^14^. Using this highly sensitive THz-TDS system, they measured the stress-caused birefringence, i.e. Δ*N*_1_ − Δ*N*_2_, when a uniaxial stress was applied on a specimen. According to their experimental result, the stress-optic coefficient *C* of PTFE was determined. Pfleger *et al*. measured the birefringence and the optical axes orientation of the LiNbO_3_ crystal^15^. But, the presented method can not be directly used for stress measurement due to relatively small magnitude of the stress induced-birefringence. Recently, we obtained the relationship between the uniaxial tensile stress and the refractive index change Δ*N*_1_ and the stress-optical coefficient *A* of PTFE^16^,^17^.

In the above works, the stress-caused refractive index changes have been experimentally measured as Δ*N*_1_ − Δ*N*_2_ or Δ*N*_1_. Based the above results, it is straight forward to measure a uniaxial stress, in which there is only one stress parameter to be measured. However, it is impossible to determine a plane stress state, in which there are three unknown parameters to be determined. Although great effort was made to characterizing birefringence in THz regime^19^,^20^, no work succeeded on determination of the plane stress state using THz wave.

In this report, we develop an experimental method to determine the plane stress state in optically opaque materials using an improved THz-TDS system. An experimental principle is also established with respect to the improved THz-TDS system. The proposed method is experimentally validated and the plane stress states of PTFE specimens were obtained.

## Methods

**Figure 1:**
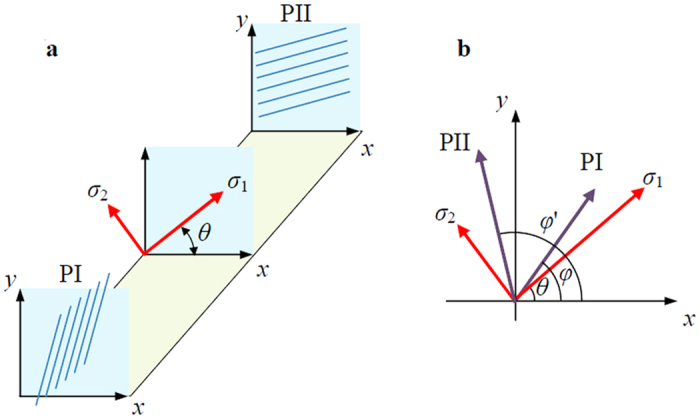
Orientation of the polarizers and the principle stress (**a**) and the projection view (**b**). PI: Polarizer I, PII: Polarizer II.

Compared with the conventional THz-TDS, the system used for plane stress state measurement is improved by integrating two polarizers to sense and adjust the polarization state of the THz waves. Figure 1 schematically shows the orientations of the two added polarizers and the stress components. In the improved THz-TDS system, the emitted THz wave is horizontally polarized. Suppose the phase and the amplitude of the emitted THz wave are 0 and 1 respectively, the electric field *E*_0_ can be expressed using the Jones vector as


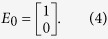


As the incident THz radiation transmits through the specimen that is loaded by a plane stress, it is divided into two polarized radiation along the two principal stress directions, which are perpendicular to each other. As illustrated in Fig. 1, the THz wave subsequently passes Polarizer I, the specimen, and Polarizer II. The received THz wave can therefore be expressed as


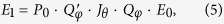


where


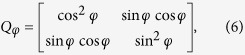



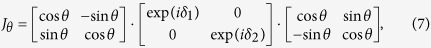



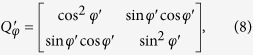


and


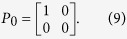


In Eq. (5), *Q*_*φ*_ and *Q*′_*φ*_ are the Jones matrix of the two polarizers, *φ* and *φ*′ are the angles between Polarizer I, II and the horizontal direction respectively, *J*_*θ*_ is the Jones matrix of the loaded specimen, *θ* is the angle between the first principal stress and the horizontal direction, *δ*_1_ and *δ*_2_ are the stress-caused phase delays of the two polarized components along the directions of *σ*_1_ and *σ*_2_, and *P*_0_ is used to extract the horizontally polarized component because the detector in the TDS system can only detect that component of the THz radiation. Equation (5) can be simplified as


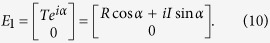


where 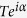 represents the received THz wave. *R* and *I* are the real and the imaginary parts, and *α* is the phase delay of the received THz wave caused by the applied stress. After simplification, the phase delay *α* can be expressed as


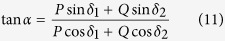


where


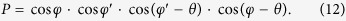



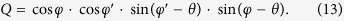


Speaking specifically, the phase delays, *δ*_1_ and *δ*_2_, are caused by the stress-optical effect and the Poisson effect when stresses are loaded on the specimen and can be calculated using the following equations as


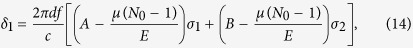



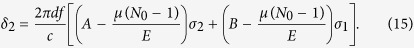


where *f* is the frequency of the THz radiation, *c* is the speed of light in air, *d* is the original thickness of the specimen, *N*_0_, *E* and *μ* are the initial refractive index, the elastic modulus and the Poisson ratio of the specimen, respectively. Therefore, the relationship between the phase delay *α* of the captured THz wave and the applied plane stress state can be determined by combining Eqs (11, 14 and 15).

To measure the three stress parameters, the two polarizers are placed at three different orientations and the phase delay data between free and loaded conditions are captured. Since the experimentally captured THz signals are broadband, the phase delay at the *n*th frequency component *f*_*n*_ for the *k*th polarizer orientation is denoted as 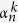. An error function of the phase delay is thereby defined as:


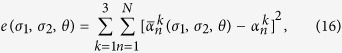


where 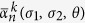 is the phase delay calculated using Eqs (11, 14 and 15) for any arbitrary set of (*σ*_1_, *σ*_2_, *θ*). The Newton-Raphson method is used to obtain the minimum of this error function. The specific value of (*σ*_1_, *σ*_2_, *θ*) that minimizes the error function is regarded as the real plane stress state undergone by the specimen.

### Experiments

Experiments were conducted on a dumbbell-shaped PTFE sheet specimen. The specimen was fixed on a mechanical loading device that provided the uniaxial tensile loading. The phase spectra of the free and loaded specimen were measured. Meanwhile, strain gauges were glued on the specimen surface to measure the tensile strain, which was used for stress calculation for comparison. Table 1 presents the geometric and physical parameters of the PTEF specimen.

In the improved THz-TDS system, two wired-grid polarizers were added to the standard THz-TDS system, where a femtosecond laser with 88 MHz repetition rate, 26 fs pulse width, and 800 nm wavelength was used as the pump source with a 10 mW average power for both the THz transmitter and receiver. The THz wave was generated and detected by shorting the dipole-type photoconductive antenna gap. Because the refractive index change to be measured would be very small, high signal stability is critical for experimental measurement. In order to keep the THz signal stable, humidity is controlled below 2% by mixing dry air into the sealed chamber during the measurement. The highly reliable frequency range of the THz-TDS system is 0.4~1.80 THz.

## Results

### Stress-optical coefficients measurement

**Figure 2:**
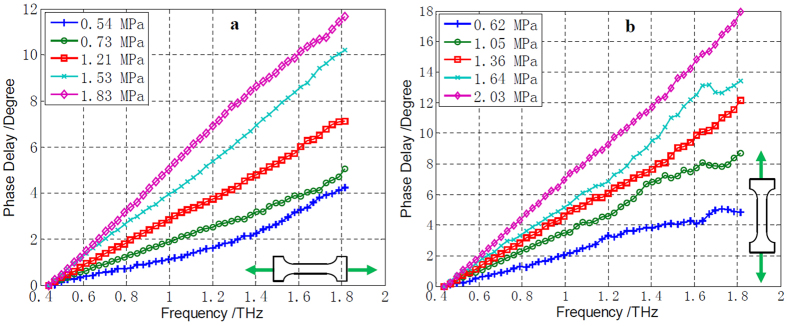
Phase delays under uniaxial stress. (**a**) Phase delays when horizontal stress loads were applied. (**b**) Phase delays when vertical stress loads were applied.

The two stress-optical coefficients *A* and *B* are imperative in stress state calculation and can only be obtained through experiments. In order to measure coefficient *A* of the PTFE specimen, the loading device was placed horizontally so that a horizontal uniaxial stress was applied on the specimen. The polarization directions of the two polarizers were also set horizontal. In this case, *θ, φ, φ*′ and *σ*_2_ are all equal to zero and *σ*_1_ is equal to the horizontally applied horizontal stress. Thus, stress-optical coefficient *A* can be obtained from Eqs (11, 12, 13, 14) as


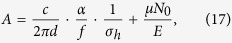


where *σ*_*h*_ is the applied horizontal uniaxial stress and *α*/*f* represents the slope of phase delay with respect to frequency when a stress is applied on the specimen. Figure 2a shows five phase delay vs. frequency curves under different horizontal stresses. The stress-optical coefficient *A* is thus obtained as 2.03 × 10^−9^ Pa^−1^ using Eq. (17).

The stress-optical coefficient *B* was similarly obtained, except that the loading device was vertically placed to obtain a vertical stress. The two polarizers were kept horizontal. In this case, *θ* = 90°, *φ* = 0, *φ*′ = 0, *σ*_2_ = 0, and *σ*_1_ equals to the vertically applied stress. The coefficient *B* could be obtained from Eqs (11, 12, 13 and 15) as


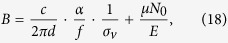


where *σ*_*v*_ is the vertically applied uniaxial stress. Figure 2b shows the phase delay vs. frequency when different vertical stresses were applied. The stress-optical coefficient *B* is then obtained as 2.63 × 10^−9^ Pa^−1^ according to Eq. (18).

### Plane stress state measurement

The plane stress state of the PTFE specimen was measured under four different loadings. In the first two experiments, horizontal loadings were applied on the specimen, while vertical loading were applied in the other two experiments. The applied stress states are obtained by strain gauge and listed in Table 2 for comparison. The specimen is supposed to be under general plane stress states. As stated previously, in order to obtain specimen plane stress state that is actually represented by the three stress parameters, *σ*_1_, *σ*_2_ and *θ*, the phase delay of the captured THz waves needs to be measured at three different orientation settings of the two polarizers. The three orientation settings in the experiment were: 1) *φ* = 0, *φ*′ = 0; 2) *φ* = 45°, *φ*′ = 45°; and 3) *φ* = 60°, *φ*′ = −60°, respectively. For each orientation setting, the THz wave was captured for both free and loading conditions. The phase delays at different frequency components were obtained by Fourier transform and served as 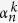 in Eq. (16).

**Figure 3:**
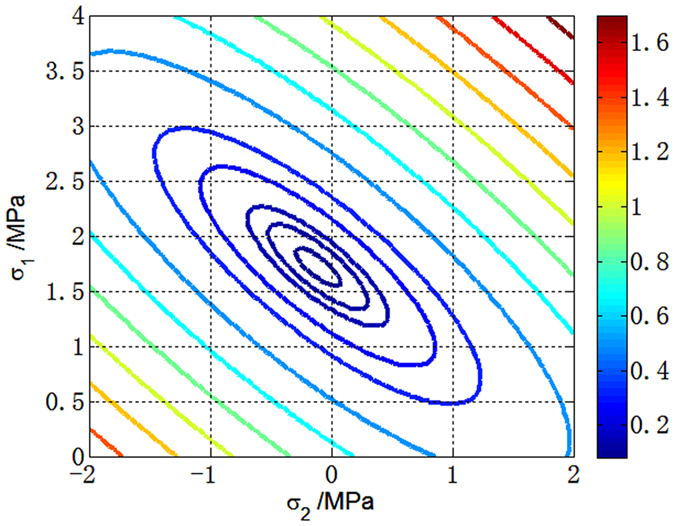
Distribution of the error function *e*(σ_1_, *σ*_2_) when *θ* = 13°.

**Figure 4:**
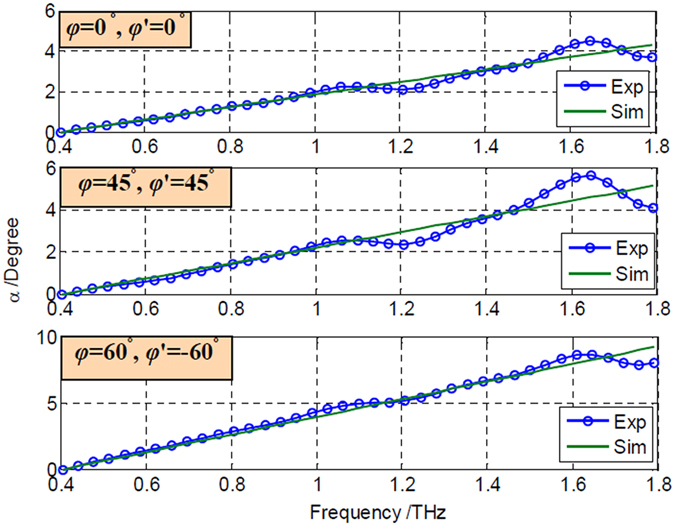
Phase delays of the first experimental measurement and theoretical simulation.

The measured plane stress states using the proposed THz method are presented in Table 2. The stress states are represented by the stress parameters that make the error function minimum under each loading condition. For the first loading condition, the plane stress state obtained by THz-TDS is *σ*_1_ = 1.7 MPa, *σ*_2_ = −0.1 MPa, and *θ* = 13°. Figure 3 shows the distribution of the error function in the *σ*_1_ − *σ*_2_ plane when *θ* = 13°. A well-shaped minimum is shown in the figure. Thus, it is reliable to achieve the minimum by the Newton-Raphson scheme. Figure 4 compares the theoretical and experimental phase delays at the frequency domain under the first plane stress state. Good agreements are indicated between the experimental results and the theoretical calculation for the three polarizer orientation settings. The error function distributions and phase delay curves of the other three measurements are provided in Supplementary Figures.

## Discussion

Comparison between the plane stress states obtained by the proposed method and the results by the strain gauges indicates a relatively apparent difference in the stress parameters *σ*_2_ and *θ*. Several factors may account for the difference. One possible reason may be the nonlinear mechanical property of PTFE. It could induce some inevitable error when strain gauges are used to measure the stress state. It is assumed in this report that free specimens are optically isotropic. But actually residual birefringence may exist in unstressed specimens. This may be another influencing factor. Stability of the THz signal also affects the precision of stress measurement. Another factor may be the relatively low extinction ratios of the metallic wire-grid THz polarizers used in the THz-TDS system. Thus, the penetrated THz waves would not be perfectly polarized. Therefore, further efforts should be contributed to improving accuracy and stability of the proposed method.

It may also be noticed that in the proposed method only the phase data of the received signal is used in stress determination, while the magnitude information is ignored, which is different or contradictory to conventional THz wave applications. This is because the amplitude data of our THz-TDS system is not stable enough. The instability of the amplitude data makes it impractical to achieve accurate measurement on stress states.

Using the proposed method, THz waves can not only be used to find interior flaw in materials, but also to determine the stress distribution when material under mechanical loading. Some flaws may be too small to be detected, but they induce obviously singular stress distributions that could be identified by stress measurement. Thus, the proposed stress measurement method here is possibly developed to be a prospective non-destructive testing method in future.

## Additional Information

**How to cite this article**: Wang, Z. *et al*. Determination of plane stress state using terahertz time-domain spectroscopy. *Sci. Rep.* **6**, 36308; doi: 10.1038/srep36308 (2016).

**Publisher’s note:** Springer Nature remains neutral with regard to jurisdictional claims in published maps and institutional affiliations.

## Supplementary Material

Supplementary Information

## Figures and Tables

**Table 1:** Physical and Geometry Parameters.

Parameters	Value
Thickness (*d*)	2 mm
Elastic Modulus (*E*)	468.5 MPa
Poisson ratio (*μ*)	0.4
Refractive index (*n*_0_)	1.417

**Table 2:** Measurement Results of the Stress State.

No.	By THz-TDS (MPa, MPa, Degree)	By strain gauge (MPa, MPa, Degree)
1	(1.7, −0.1, 13)	(1.7, 0, 0)
2	(2.2, −0.7, −14)	(2.5, 0, 0)
3	(1.8, −0.5, 79)	(1.7, 0, 90)
4	(2.4, 0.2, 114)	(2.5, 0, 90)
