# Supplementary material for: Determination of plane stress state using terahertz time-domain spectroscopy
Source: Sci Rep. 2016 Nov 8;6:36308. doi: 10.1038/srep36308 (PMC5099881; doi:10.1038/srep36308)

Supplementary Information

Determination of plane stress state using terahertz time-domain spectroscopy

Zhiyong Wang, Kai Kang, Shibin Wang, Lin′an Li, Ningning Xu, Jiaguang Han, Mingxia He, Liang Wu and Weili Zhang

SUPPLEMENTARY FIGURES

Supplementary Figure 1

The error function distribution of the second experimental measurement $e(\sigma_1, \sigma_2)$ when θ=-14°

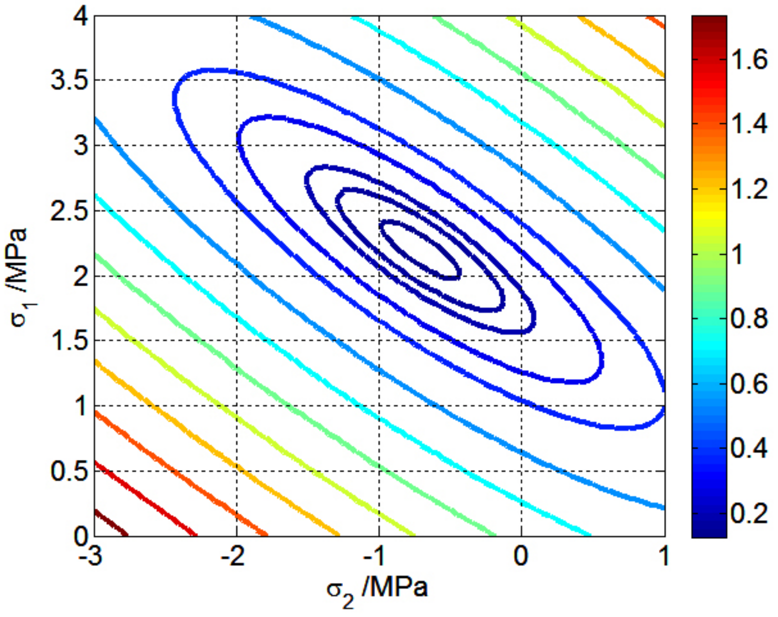

Supplementary Figure 2

The error function distribution of the second experimental measurement $e(\sigma_1, \theta)$ when σ_2=-0.7MPa

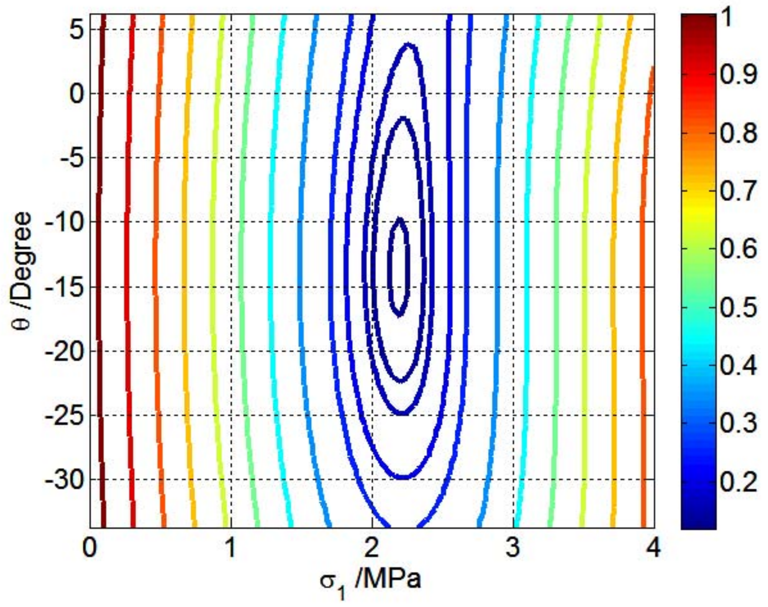

Supplementary Figure 3

The error function distribution of the second experimental measurement $e(\sigma_2, \theta)$ when σ_1=2.4MPa

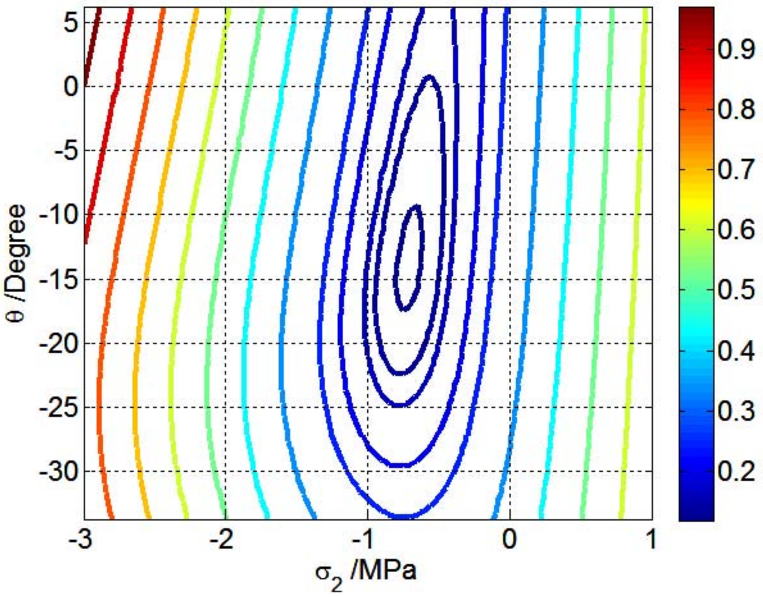

Supplementary Figure 4

The phase delays of the second experimental measurement and theoretical simulation

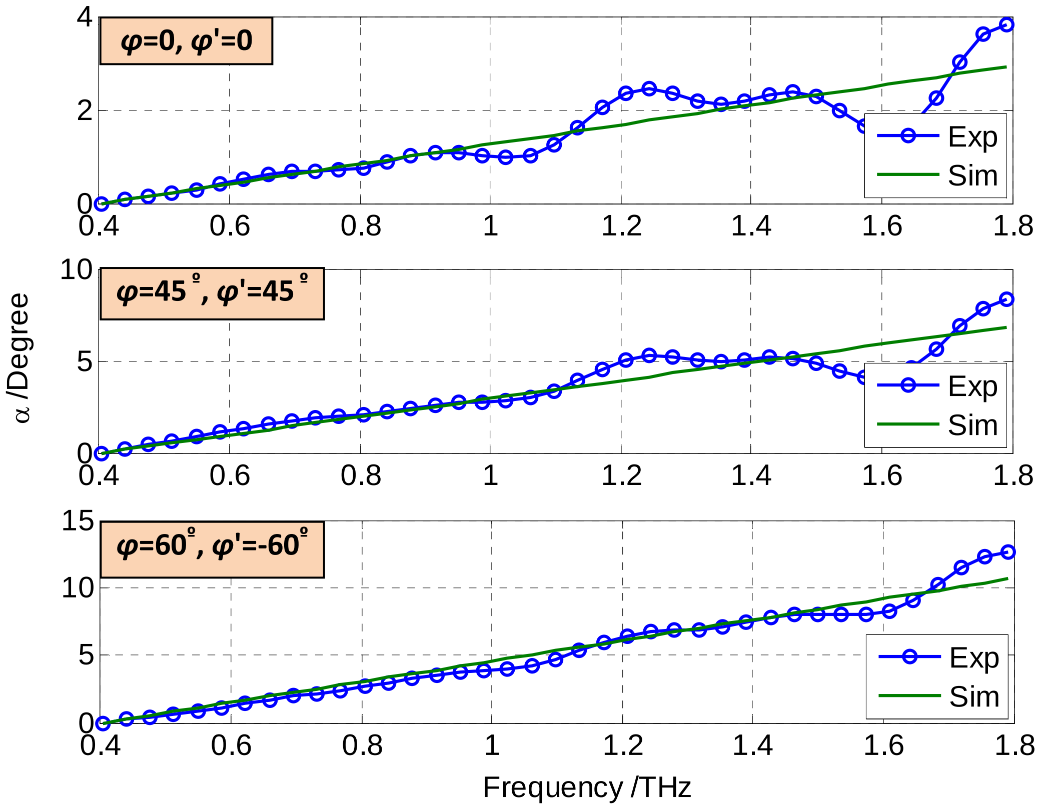

Supplementary Figure 5

The error function distribution of the third experimental measurement $e(\sigma_1, \sigma_2)$ when θ=79°

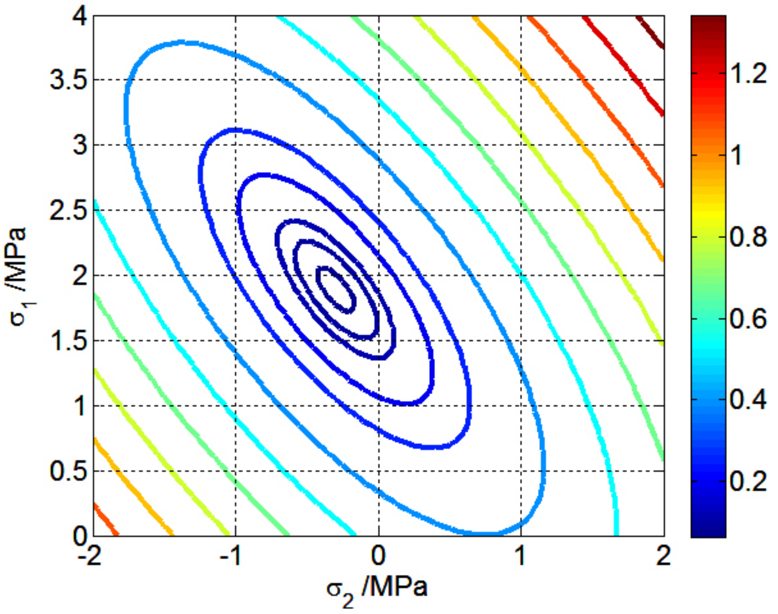

Supplementary Figure 6

The error function distribution of the third experimental measurement $e(\sigma_1, \theta)$ when σ_2=-0.5MPa

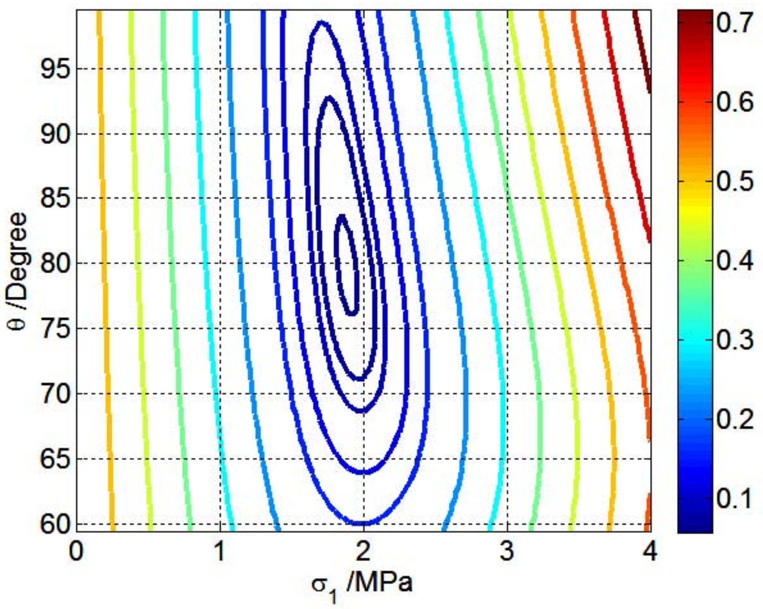

Supplementary Figure 7

The error function distribution of the third experimental measurement $e(\sigma_2, \theta)$ when σ_1=1.8MPa

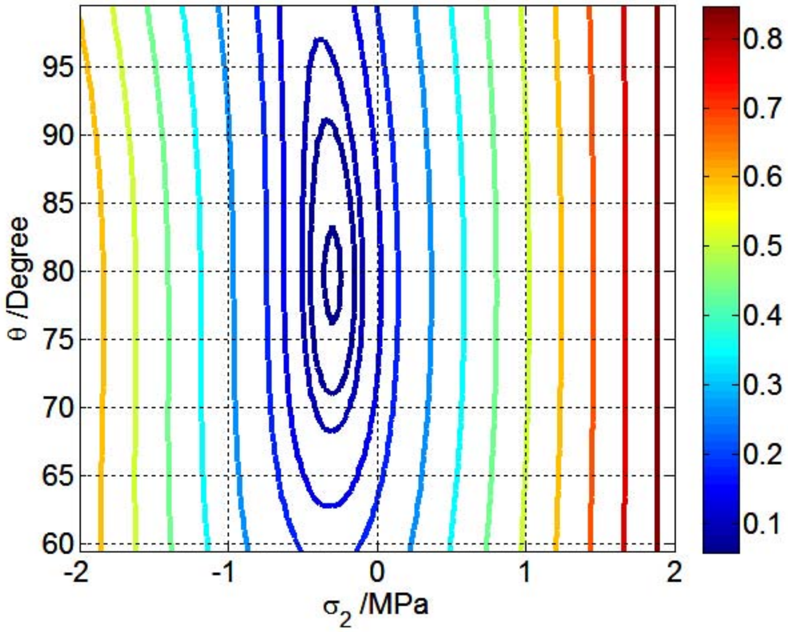

Supplementary Figure 8

The phase delays of the third experimental measurement and theoretical simulation

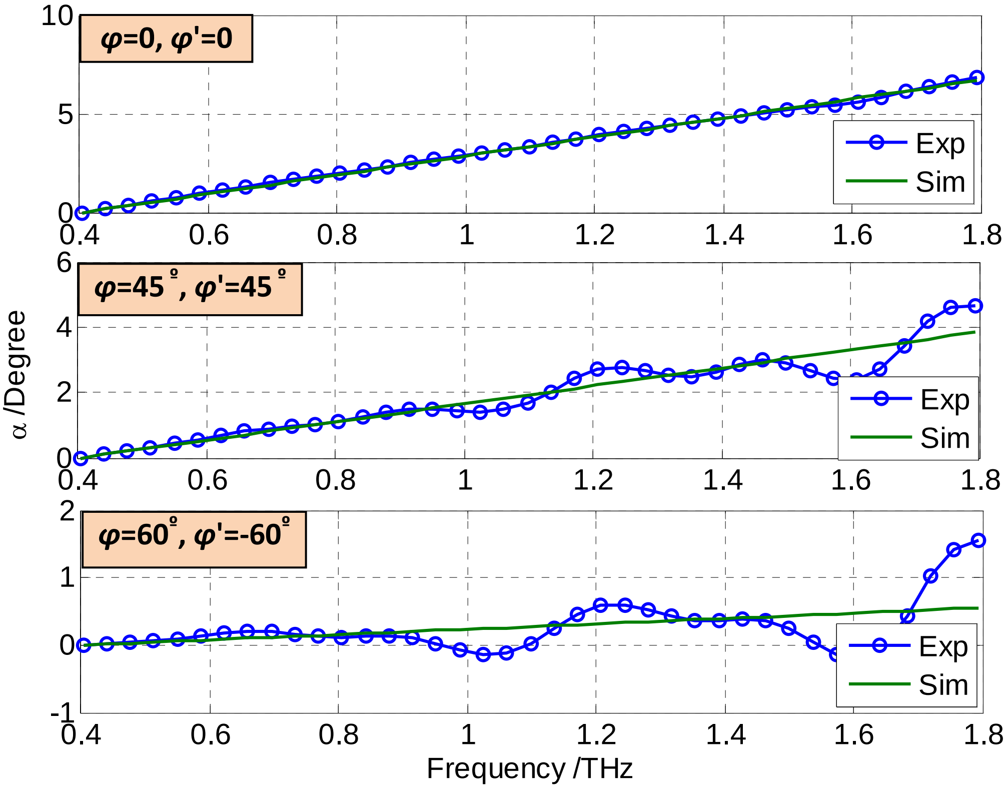

Supplementary Figure 9

The error function distribution of the fourth experimental measurement $e(\sigma_1, \sigma_2)$ when θ=114°

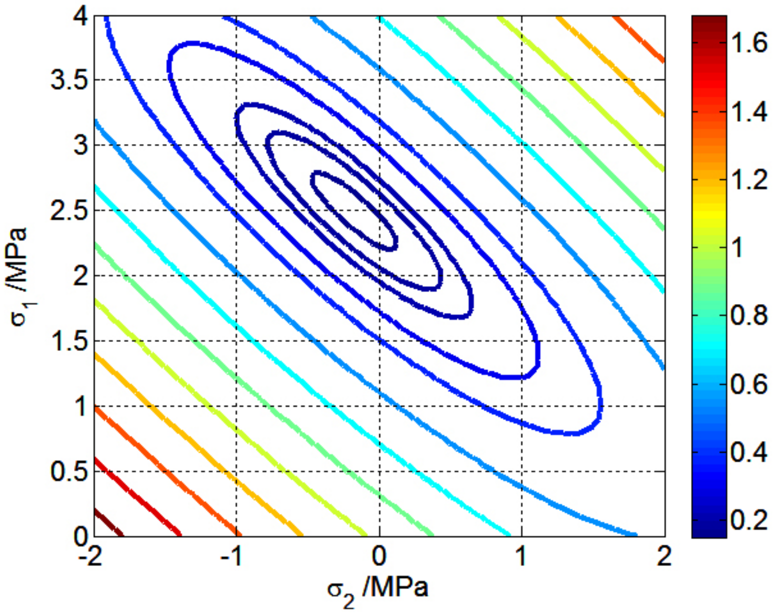

Supplementary Figure 10

The error function distribution of the fourth experimental measurement $e(\sigma_1, \theta)$ when σ_2=0.2MPa

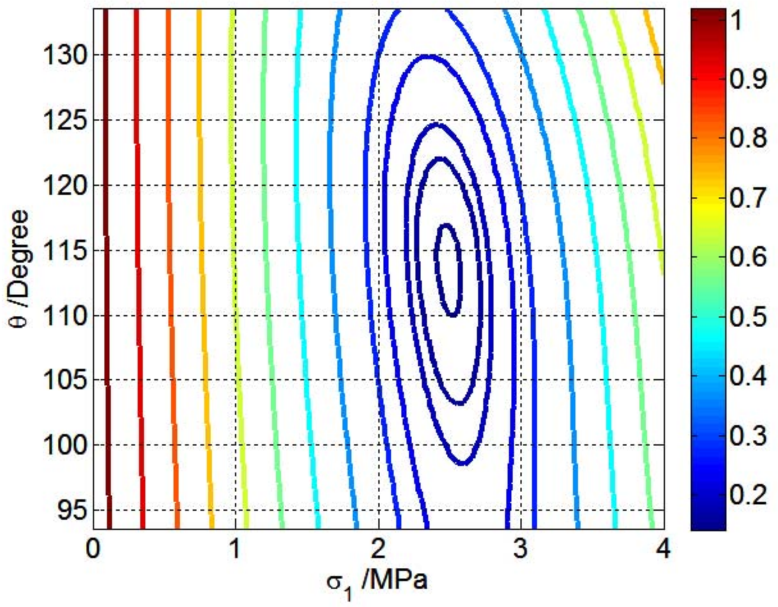

Supplementary Figure 11

The error function distribution of the fourth experimental measurement $e(\sigma_2, \theta)$ when σ_1=2.5MPa

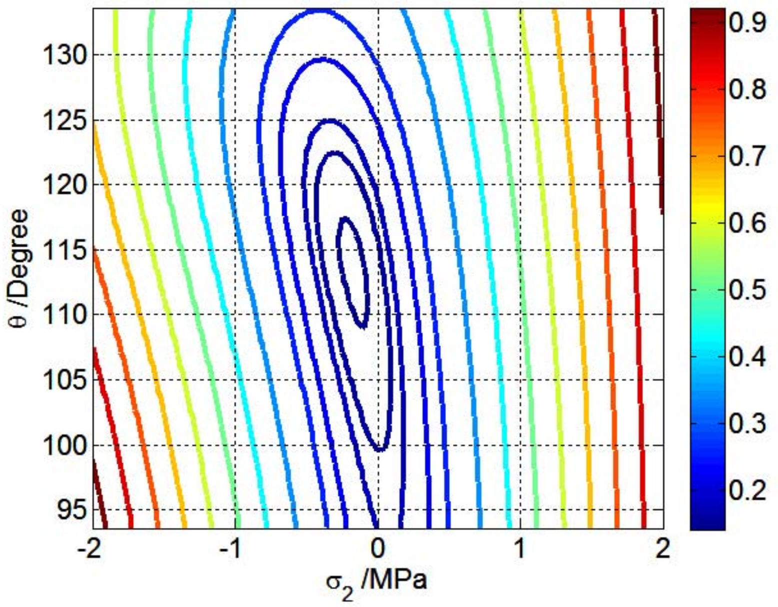

Supplementary Figure 12

The phase delays of the fourth experimental measurement and theoretical simulation

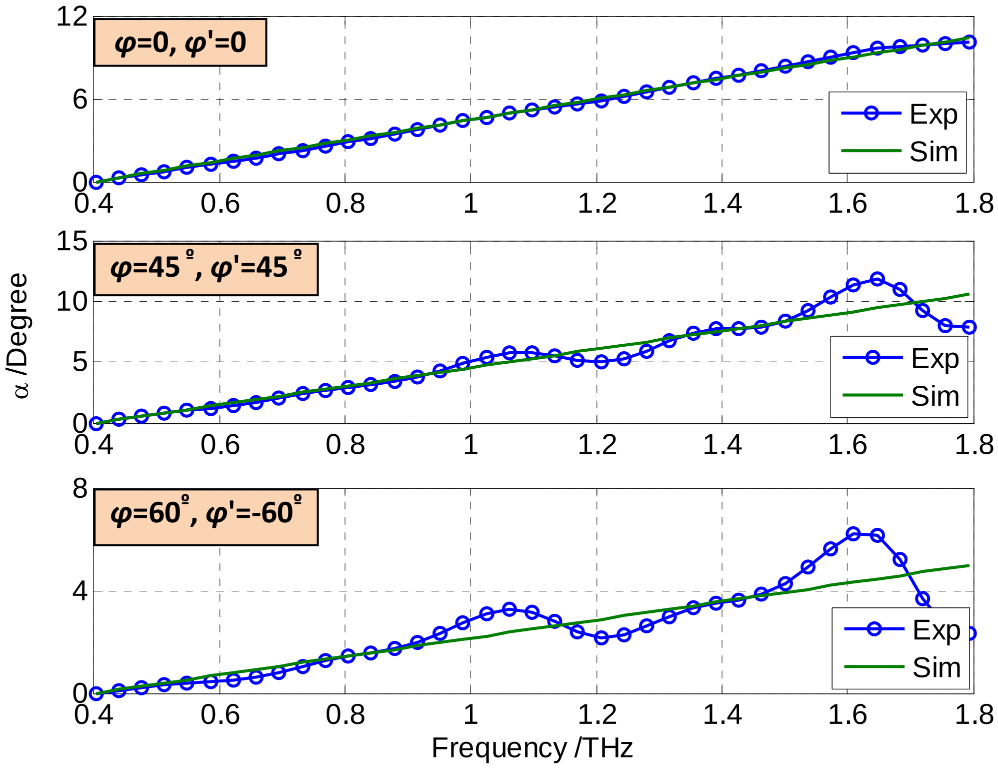

Supplement: Supplementary Information [file srep36308-s1.pdf]
